# Inclusion of emerging organic contaminants in groundwater monitoring plans

**DOI:** 10.1016/j.mex.2016.05.008

**Published:** 2016-05-25

**Authors:** Lucrezia Lamastra, Matteo Balderacchi, Marco Trevisan

**Affiliations:** Università Cattolica del Sacro Cuore, Istituto di Chimica Agraria ed Ambientale, via Emilia Parmense, 84, 29122 Piacenza, Italy

**Keywords:** Inclusion of emerging organic contaminants in groundwater monitoring plans, Groundwater, Pollution, Monitoring, Priority list, Emerging pollutants, Environmental tracers, Organic wastewater contaminants

## Abstract

Groundwater is essential for human life and its protection is a goal for the European policies. All the anthropogenic activities could impact on water quality.

• Conventional pollutants and more than 700 emerging pollutants, resulting from point and diffuse source contamination, threat the aquatic ecosystem.
• Policy-makers and scientists will have to cooperate to create an initial groundwater emerging pollutant priority list, to answer at consumer demands for safety and to the lack of conceptual models for emerging pollutants in groundwater.
• Among the emerging contaminants and pollutants this paper focuses on organic wastewater contaminants (OWCs) mainly released into the environment by domestic households, industry, hospitals and agriculture. This paper starts from the current regulatory framework and from the literature overview to explain how the missing conceptual model for OWCs could be developed.
• A full understanding of the mechanisms leading to the contamination and the evidence of the contamination must be the foundation of the conceptual model. In this paper carbamazepine, galaxolide and sulfamethozale, between the OWCs, are proposed as “environmental tracers” to identify sources and pathways ofcontamination/pollution.

## Introduction

Following the prevision of the United Nations by 2050 the world’s population will reach 9.6 billion [107]. This population rise will be supported by an increase of the agricultural and industrial activities that will produce a greater water stress due to an increased demand for freshwater and to an increased generation of wastewater. Groundwater pollution by anthropogenic activities is a threat to human and ecosystem health and wellbeing, in fact groundwater is a source of fresh water for human consumption, irrigation and ecosystem needs, and its protection is a key environmental objective. In addition to the known pollutants, new substances with no clear immediate effects are emerging [37]. It is important to be aware of these new pollutants in monitoring programmes and in developing groundwater protection policies, because their effects can affect coming generations [96]. Until now, water quality legislation has not systematically dealt with emerging pollutants in groundwater for several reasons, including a lack of knowledge of contaminant sources and pathways, properties and effects of substances and analytical detection techniques. In the last years the advances in analytical chemistry allowed the detection of chemicals in water bodies at very low concentrations [69]. The use of high resolution mass spectrometers like the QTOF technology, coupled with multiresidues methods help to perform target and non-target screening followed by quantitative determination [85]. Emerging contaminants could be natural or synthetic substances that are not commonly monitored in the environment [102]. They can encompass chemicals not previously included in national or international monitoring programmes but continuously introduced into the environment by anthropogenic activities [90], and well-known contaminants that have gained interest with the revelation of new aspects of their occurrence, fate or effects [22]. Accordingly to Geissen et al. [42] more than 700 emerging pollutants, their metabolites and transformation products are listed as present in the European aquatic environment (www.norman-network.net). The fact that emerging pollutants are present in water bodies as complex mixture has to be considered. The ubiquity and the high number of potentially toxic compounds could lead to synergistic effects [85].

Contaminants, pollutants, indicators and environmental tracers could reach groundwater bodies. Contaminants are substances present in places where they should not be, or at concentrations above background [15]. Pollutants are contaminants that result in, or can result in, adverse biological effects [15]. Indicators are measured or observed substance properties, or values derived from these, which describe the state of a phenomenon/environment/area, with a significance extending beyond that directly associated with a parameter value [78]. Environmental tracers are detectable material accidentally present or added in small quantities to flowing surface water or groundwater, depicting the pathways or serving in the measurement of flow characteristics.

The identification of sources and pathways of contamination/pollution and the prediction of their impacts on groundwater quality are possible combining indicators and tracers. This is useful for the development or the improvement of new conceptual models. Conceptual models intend to describe and optionally quantify systems, processes and their interactions [36] and are developed to different incremental degrees of complexity. Emerging contaminants and pollutants include any compound for which a conceptual model is missing. A way to develop management strategies without a conceptual model for the emerging pollutants is to consider their sources of contamination. The presence of emerging pollutant in water bodies traditionally could be the result of point (mainly urban and industry) or diffuse (agriculture) pollution. Non-point source pollution usually regards large areas and may cause larger impact on groundwater quality than point-source [52]

**Fig. 1:**
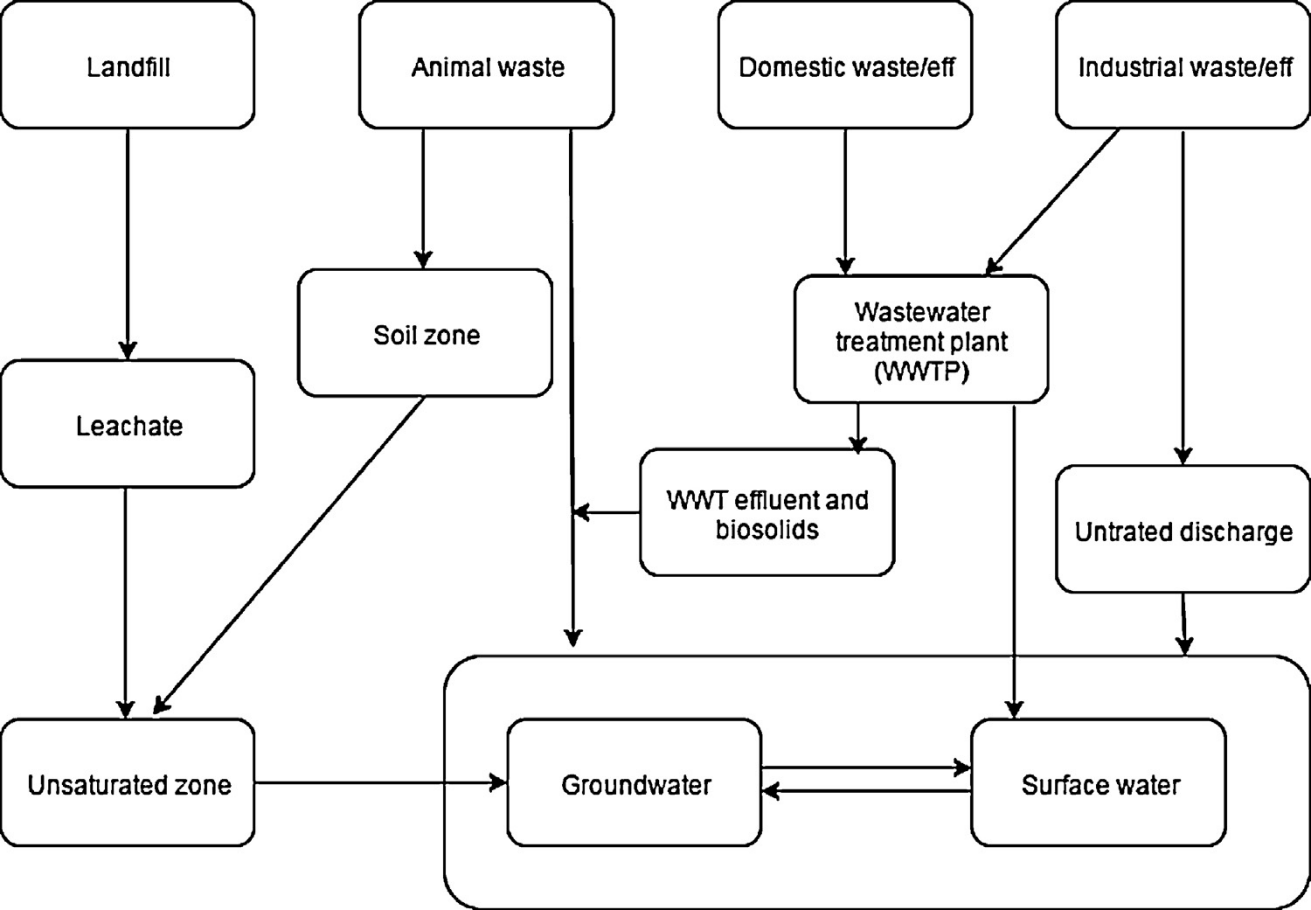
The major pathways of contamination of OWCs from their sources to groundwater (from Lapworth et al. [60], revisited).

In this paper, Organic Wastewater Contaminants (OWCs, Table 1) are used as an example. OWCs can include pharmaceutical products, industrial compounds, pesticides and other emerging pollutants (personal care, life style and cosmetics products etc.). In terms of chemical use and emissions, pesticide use and agriculture sector are one of the main responsible of the diffuse pollution [42]. Anyway the contamination profile is dominated by industrial compounds, followed by pesticides and pharmaceuticals [52]. OWCs are primarily released into the environment by domestic households, industry, hospitals and agriculture (Fig. 1), while secondary contamination of soils and vegetation can occur through utilisation of biosolids, sludge and manure in agriculture [99]. Other specific sources of OWCs in groundwater are sewer leaching and urban storm water recharge, both of which directly affect urban groundwater. Moreover, these contaminants are present in the effluents from wastewater treatment plants and can contaminate rivers and through-flow lakes. OWCs may also be introduced into karstic groundwater through disposal of partly treated sewage to sinkholes and streams [32]. Another pathway by which OWCs reach groundwater relates to wastewater management, namely the conditioning and re-use of greywater [68]. The pathways of contamination from source to groundwater have already been identified (Fig. 1), but in-depth conceptual models of OWCs in the groundwater are still lacking.

Not only the source of contamination, but also dilution, adsorption, transformation and degradation rate can affect the concentration of OWCs in the groundwater. Moreover the transport of the pollutants to the water body is depending on their volatility, polarity, persistence, and adsorption properties.

Because of consumer concerns about safety, the high number of potentially monitored compounds, the high cost of monitoring and the scarcity of data on the effects and behaviour of emerging pollutants, a strategy for prioritising substance monitoring is necessary.

The aim of this paper is to give advice on the investigative monitoring of groundwater, because research on OWCs and emerging pollutants in groundwater are needed for threshold of regulation in order to obtain reference values to evaluate the water quality and to ensure human health. Moreover often the produced literature on this concern presents results difficult to compare, in fact in similar studies there is a disparity of the targeted compounds due to the different analytical methodologies and capabilities and to the different criteria used to select chemicals and sampling sites [63]. In addition the few monitoring studies that considers large scale are mono compartmental [42], and make difficult to obtain the full picture of the exchange and transformation dynamics between the involved compartments without a conceptual model.

## Current regulatory framework

There are lists of priority or regulated contaminants (at least for the USA and EU), but there is no clearly stated procedure to identify compounds which should be included in monitoring programmes. However, several authors published approaches for establishing priority lists with different levels of complexity.

The US Safe Drinking Water Act (SDWA) includes a process to identify and list unregulated contaminants that may require a national drinking water regulation in the future. The US Environmental Protection Agency (USEPA) periodically publishes this list of contaminants (called the Contaminant Candidate List or CCL) and decides whether to regulate new contaminants on the list (called Regulatory Determinations). USEPA uses a health effects occurrence-analytical methods-based multi-step process to identify contaminants for inclusion in this list. Starting from 7500 potential chemical and microbial contaminants, 60 contaminants were selected in the first CCL (CCL1), 51 contaminants in CCL2 and 104 chemicals or chemical groups (chemicals used in commerce, pesticides and disinfection by-products) and 12 microbiological contaminants (waterborne pathogens and biological toxins) in CCL3.

The European Directive on pollution caused by certain dangerous substances discharged into the aquatic environment [30] (2006/11/EC) establishes at Member State level a list of substances for preventing (List 1) and a list of substances for limiting (List 2) their introduction into the aquatic system. List 1 is based on toxicity, persistence and bioaccumulation of compounds and includes organ halogens, organophosphorus compounds and carcinogenic substances. List 2 is based on harmful effects to groundwater and also comprises biocides, substances with effect on taste or smell, cyanides and fluorides. The European directive on the protection of groundwater against pollution and deterioration (GWD-Groundwater Directive (2006/118/EC)) sets groundwater quality standards and introduces measures to prevent or limit inputs of pollutants into groundwater. Moreover the directive sets quality criteria based on local characteristics. Further improvements are allowed to be made based on monitoring results and on new scientific knowledge. The Drinking Water [31] Directive (98/83/EC- directive on the quality of water intended for human consumption) concerns the quality of water intended for human consumption. The directive sets the essential quality standards at EU level including 48 microbiological, chemical and indicator parameters that have to be regularly monitored and tested. The European Water Framework Directive (WFD; 2000/60/EC) considers fresh and ground waters as a continuum and leaves many choices open to Member States. A Technical Group on Groundwater was established in the framework of Common Implementation Strategy (CIS) to provide a general scheme for contaminant inclusion in monitoring programmes. The first step of the WFD was to establish by way of Decision 2455/2001/EC a first list of priority substances, this first list was replaced by Annex II of the Directive on Environmental Quality Standards [29] Directive 2008/105/EC) (EQSD), also known as the Priority Substances Directive, which set environmental quality standards (EQS) for the substances in surface waters (river, lake, transitional and coastal) and confirmed their designation as priority or priority hazardous substances, the latter being a subset of particular concern. As required by the WFD and EQSD, the Commission subsequently reviewed the list and in 2012 made proposal for a Directive amending the WFD and the EQSD as regards priority substances. The directive 2013/39/EU introduces a list of compounds, in 2014 the list has been completed adding twelve new substances of which three drugs: diclofenac, 17-beta-estradiol, and 17-alpha-ethinylestradiol European commission, 2012. On 2015, a report released by the JRC [51] proposed other seven substances and analytical methods to monitor them. The seven substances have been selected considering the risk quotient, the information gaps, and the “emerging” pollutants. Each substance was selected based on an assessment of the exposure, hazard and risk involved, and the lack of monitoring data at European level. The seven selected compounds/class of compounds are: oxadiazon; methiocarb; 2,6-ditert-butyl-4-methylphenol; triallate; neonicotinoids (imidacloprid, thiacloprid, thiamethoxam, clothianidin, acetamiprid); antimicrobials (erythromycin, clarithromycin, azithromycin); 2-ethylhexyl-4-methoxycinnamate.

## Inclusion in a monitoring programme

Three monitoring approaches are identified in the EU Water Framework [28] Directive (WFD; 2000/60/EC): surveillance, operational and investigative monitoring. The first one assesses long-term changes due to anthropic activity, the second establishes the status of groundwater bodies or groups of bodies determined as being at risk and assesses any changes, and the third identifies problems arising from the first two. However, the first two activities require the existence of a conceptual model and therefore the only possible monitoring approach in the case of OWCs is an investigative monitoring.

A methodology for distinguishing pollutants from contaminants and for giving priority to pollutants is needed in order to plan investigative monitoring programmes and meet consumer demands on safety. In fact, the majority of OWCs do not place human health at risk but, continued vigilance in assessing the significance and implications of ‘emerging’ contaminants is necessary to support and ensure the long-term sustainability and security [19].

Following a risk assessment approach four steps have been identified [1] and can be used for setting a priority list in the absence of a conceptual model: (1) problem formulation, (2) hazard characterisation (exposure-response assessment), (3) exposure characterisation (assessment), and (4) risk characterisation.

### Problem formulation

Problem formulation is the process by which assessment objectives are developed into an assessment strategy, including the drafting of appropriate assessment (effect) endpoints. Four principles (pollution prevention, ecological threshold, community recovery and functional redundancy) are needed for establishing protection goals and can be used as a starting point [9]. In this key step, policy-makers and scientists have to cooperate for the establishment of clear goals: government authorities (policy-makers) have to set criteria for protecting water and life, balancing between economic and ecological consequences [9], while scientists have to support policy with scientific data and assist the policy-makers in their decisions [88]. USEPA bases its regulations on the projected adverse health effects from the pollutant, the extent of its occurrence in drinking water, and whether regulation of the pollutant would present a ‘meaningful opportunity’ for reducing risks to health.

The components of the European Water Framework Directive (WFD) dealing with groundwater were developed for achieving good quantitative and chemical status of groundwater. Groundwater Directive (GWD) represented a scientific response to the requirements of the Water Framework Directive (WFD) as it is related to the assessment of chemical status of groundwater and the identification of significant trends in pollutant concentrations. WFD requires that management is carried out within a river basin district to protect ecosystems, drinking water and bathing water. WFD takes into account pressures and impacts of human activity on groundwater status. Taken together, these should ensure the protection of groundwater from all contamination, according to the principle of minimum anthropogenic impact.

### Hazard characterisation

Hazards can be characterized by several endpoints related to different organisms (human and non-human), according to the objective of the assessment. Among these reference values, the most important are the acute and chronic toxicity (LDx and LCx), the no effect concentration (NOEC) and the allowed daily intake (ADI). It is also important to consider the synergistic or additive effects of substances with the same mechanism of action. However, the identification of pollutants from contaminants cannot be done solely on the basis of chemical analyses, because such analyses provide no information on bioavailability or toxicity. Effects-based measures such as laboratory or field toxicity tests and measures of the status of resident and exposed communities provide key information but cannot be used independently to determine pollution status [15]. When working with OWCs and emerging pollutants, a further issue arises: the scarcity of toxicity and ecotoxicity information for most of the compounds [7], [97]. For this reason, the endpoints for OWCs are often estimated using quantitative structure-activity relationship (QSAR) approaches. In the last years Intelligent Testing Strategies have been used including integration of complementary methodologies like QSAR, read-across models, threshold of toxicological concern, exposure information, in vitro testing methodologies, and other computational models Geisser et al., 2015. In addition physiologically based pharmacokinetic models are used to describe the biodistribution of chemicals [42].

### Exposure characterisation

In the case of OWCs, exposure characterisation is complex because of the scarcity of data on both environmental exposure and response, which prevents the development of conceptual models. For this reason, the consumption or use information for a specific OWC or the frequency of detection and maximum environmental concentration are often used. More complex approaches take into account the human metabolism or the efficiency of different wastewater treatment plants, or assess the OWCs concentration in the environment.

### Risk characterisation

Risk characterisation, which integrates the information coming from the first three steps, is the quantitative analysis of the exposures and the effects. It is used for describing the risk and for informing and supporting risk management objectives and decisions [1]. Several tools are available and are characterized by different levels of complexity and objectivity. A few are only descriptive (i.e. checklist, matrices), while others use mathematical models and can be incorporated into software. OWCs are a wide class of compounds characterized by a lack of information about exposure and effects, so a pragmatic approach giving priority to the compounds that have to be included in the monitoring programme is necessary. Ranking and scoring systems (RSSs) are a category of Decision Support Systems software developed for screening. RSSs do not provide a measure of hazard or risk but help to determine the potential for a chemical to cause environmental effects based on what we know about its persistence, bioaccumulation and toxicity [71], [110].

In the literature, different approaches have been developed for creating a priority list for surface water, but no explicit ranking system has been established for OWCs in groundwater (Table 2). The easiest approaches rely solely on assessment of the exposure or the toxicity, or on the availability of analytical techniques. In a preliminary study, [27] developed a screening system for OWCs that selected as an indicator of contamination any compounds occurring at a frequency of above 80% and present in secondary- or tertiary-treated wastewater at concentrations at least five times higher than their respective limits of quantification. [100] gave priority to pesticides with high dosage, wide usage and low K_OC_. [65] made a province-scale priority index for the monitoring of pesticides in different environmental matrices based on sales, degradation and fugacity properties. [77] ranked chemicals according to structure (QSAR approach) and expected fish toxicity. [13] used endocrine disruption potential, and [95] ranked according to toxicity for algae or/and daphnia or/and fish or/and by K_ow_ and removal efficiency in WWTP. [21] ranked pharmaceuticals using five different combinations of physical–chemical and toxicological data.

More complex approaches couple exposure and toxicity. The [33] guideline for environmental risk assessment of medicinal products for human use established a pre-screening (log K_ow_ > 4.5) and a two-tier procedure. In the first step, maximum daily dose consumed per inhabitant, fraction of market penetration, amount of wastewater produced per capita and dilution factor are used for calculating the concentration in surface water. If the predicted concentration is higher than 0.01 μg L^−1^, a second step is introduced and environmental fate and effect analysis is performed. Christen et al. [17] refined the EMEA approach by prioritising highly active compounds, i.e. compounds active at low doses, with a specific mode of action and active in important metabolic pathways. Schriks et al. [97] developed a stepwise system considering only the substances with log K_ow_ < 3 based on the ratio between drinking water guideline values and the maximum concentration detected in water reported in the literature. [84] developed a stepwise system for pharmaceuticals where all the compounds included in the Kümmerer list [57] or with log K_ow_ > 3 were considered because of their mode of action or potential for bioaccumulation. The compounds not included in this first list were screened according to their probability of reaching the open environment. Clarke and Smith [19] developed an assessment matrix for selected organic contaminants in sewage sludge based on the environmental persistence in soil (>6 months), the potential for human health impacts resulting from the application of bio solids to land, the evidence or likelihood of bioaccumulation in humans or in the environment, the evidence of ecotoxicity, and the quality of empirical data and trends on the contaminant in bio solids. The EURAM procedure [46] assesses the risk to the environment and consumers using a simple exposure-effect model.

Other authors tried to overcome pure risk characterisation. Eriksson et al. [34] identified a list of 12 organic priority pollutants for storm water by the Chemical Hazard Identification and Assessment Tool (CHIAT; [7]. This tool is based on a hazard assessment that involves the identification of receptors and exposure pathways, leading to estimation of risk quotients but asking the collaboration of stakeholders. De Voogt et al. [24] ranked pharmaceutical screening reports and papers, identifying seven criteria (regulation, consumption, physical-chemical properties, degradability, and resistance to treatment, toxicity and ecotoxicity) and scoring the compounds according to those criteria. Muñoz et al. [75] developed a LCA-based ranking system for organic wastewater contaminants identifying 16 priority compounds. Arnot and Mackay [2] developed a multi-criteria risk assessment tool, while Kumar and Xagoraraki [56] proposed a multi-attribute approach based on occurrence, treatment efficiency, ecological effects and health effects. Ortiz de Garcìa et al. [79] proposed a ranking of pharmaceuticals considering chemical properties, human consumption, metabolism, predicted environmental concentrations and human and environmental toxicities. In 2015 JRC proposed a restricted number of substances (up to 10) to be included in a dynamic Watch List, remaining there for limited time. The substances identified for inclusion were selected based on the suspected risk to or via the aquatic environment, as well as on the unavailability of sufficient monitoring data or data of sufficient quality to identify the risk posed by those substances, and to prioritise them at EU level.

The development of a ranking system for groundwater is possible and expected, but the consumer demands on safety can be satisfied and pollution can be prevented only by ranking systems that consider both exposure and effects. The exposure assessment should not be limited to human or to ecosystem receptors. The model-based tools for predicting contaminant concentration in groundwater (i.e. EURAM, EMEA) should be preferred because they allow a reduction of the uncertainty in a transparent and scientifically sound manner [15]. Unlike the case for surface water, the contamination paths to groundwater are not sufficiently well described. Therefore, pragmatic approaches (i.e. [56]) that consider consumption/use, measured concentration and detection frequency can be used in setting up an investigative monitoring programme.

## Investigative monitoring and creation of a conceptual model

As reported by Balderacchi et al. [3] the Driver, Pressure, State, Impact, Response (DPSIR) analytical framework is commonly used for identifying impacts and pressures in order to attain the goal of good groundwater status in Europe [20]. DPSIR fails for the new or emerging compounds. In fact DPSIR is deductive and requires a conceptual model and the definition of dependencies among its elements. Therefore monitoring approaches and indicators of contamination are required in order to propose improved monitoring plans that combine physical, chemical and biological indicators and combine science with policy.

Because of the absence of a conceptual model, the monitoring of groundwater should reflect potential OWCs contamination sources and patterns. The first attempts will focus on simplified paths: from households to WWTPs to rivers and to groundwater; and from bio solids, sludge and greywater to soils and groundwater [4]. The importance of identifying the factors that are the most important in determining the occurrence and concentrations of OWCs in groundwater or that allow the identification of possible ‘hot spot’ areas of pollution, was already emphasised in the two largest monitoring studies to date [5], [62]. In the second step, conceptual models will also take into account sewer leaching and urban storm water recharge.

Environmental tracers can provide valuable information on natural attenuation of dissolved organic contaminants in groundwater systems. Persistent organic compounds themselves can be used to trace contaminated flow through aquifers [47], [67], [32]. The conceptual and methodological frameworks for the application of environmental tracers in studies on the presence and fate of OWCs in groundwater systems are therefore not different from the well-established principles of tracing groundwater transport processes. Such application of environmental tracers can be considered in a broader perspective as contributing to the development of conceptual models of groundwater bodies threatened by OWCs contamination. Use of environmental tracers enhances the capability of conceptual models by providing time scales of solute transport [76]. Knowledge of contaminant transit time distributions allows for estimation of: (i) time lags associated with responses of the system to both commencement and cessation of contamination and (ii) maximum concentration of contaminants at discharge areas.

Based on these considerations Balderacchi et al. [4] suggested introducing tetrachloroethylene, perchloroethylene, and trichloroethylene in the EQS list of the improved annex I of the GWD. These emerging contaminants, in fact, are persistent ubiquitous in extensive area in Europe, and the most prevalent organic contaminants found in groundwater, and could be selected as indicators of the urban sprawl.

Other environmental tracers could be used in the investigative monitoring in order to achieve the conceptual model. The selection of an environmental tracer of the OWCs has to be done considering that OWCs could be indicators of diffuse and point pressure from household, dump and storage sites and following these principles:

- The selected compound is organic.
- The selected compound has been found in the groundwater.
- The selected compound is characterized by high toxicity.
- The selected compound is characterized by low removal rates in the WWTP.

Considering the most commonly OWCs as presented by Balderacchi et al. and considering information on the presence of some of the same OWCs collected from extensive literature studies a priority ranking could be defined. In order to do this only the OWCs revealed with frequency higher than 20% have been selected and ordered following the frequency of detection, from the higher to the lower. Maximum concentration in GW was also considered, and the selected OWCs have been ordered following maximum concentration value. The OWCs have been assigned to a toxicity class considering the value of LD_50_ oral (rat). Three classes have been defined: high toxicity (LD_50_ lower than 1000 mg/kg bw) medium toxicity (LD_50_ between 1000 mg/kg bw and 5000 mg/kg bw) and low toxicity (LD_50_ higher than 5000 mg/kg bw). Finally the removal from the wastewater treatment plant (WWTP) has been considered; also in this case three classes have been defined: low, medium and high removal. An overall ranking could be constructed following the four rankings based on frequency, maximum concentration in GW, toxicity and removal classes (Table 3).

Pharmaceuticals and personal care products are some of the OWCs often found in the wastewater. Moreover very few synthetic studies exist on the removal of emerging pollutants during treatment in wastewater treatment plant (WWTP). Wastewater treatments, in fact, are necessary to eliminate potential toxic compounds but their efficiency is not yet clearly known, and they were not originally designed for elimination of xenobiotics [25].

Drugs with different chemical structure undergo different fate in the WWTP, for instance the lowest removal rate is reported for the class of antiepileptics and the highest for antidepressants (with over 90%) [25]. Carbamazepine, an antiepileptic, has a removal rate of −5, 7% indicating a non-removal. Due to this fact and due to its refractory nature carbamazepine is one of the most commonly identified compounds. In a pan-European survey carbamazepine was found in 42% of the selected ground-water samples. A likely reason of these results could be also attributed to the widely prescription and purchase over-the-counter (>1000 kg per annum; [85]. Moreover, carbamazepine was detected in tile drainage from either field section prior to bio solid application. Carbamazepine has been considered by several authors useful source-specific tracer of domestic wastewater contamination [50] (Tables 4–6 ).

In the cosmetic class synthetic musks are used as fragrances in a wide range of washing and cleaning agents and personal care products. Among them galaxolide (1,3,4,6,7,8-hexahydro-4,6,6′,7,8,8′-hexamethylcyclopenta-[γ]-2-benzopyran; HHCB) and tonalide (7-acetyl-1,1′,3,4,4′,6-hexamethyl-1,2,3,4-tetrahydronaphthalene-AHTN) are the most important commercial synthetic musks and their use accounts for 95% of the total market volume of polycyclic musks, being estimated in Europe at 358 ton/y and at 1473 ton/y in 2000, respectively [82]. These compounds are characterized by medium-high removal (40–90%) [61] in wastewater treatment plants, but, anyway they have been sporadically detected in groundwater [102], [80], [105]. Galaxolide and tonalide have been detected in some European rivers (Table 5). They often occur in water and groundwater located near wastewater discharge areas, with peak environmental concentrations occurring near effluent discharge points (Tables 4–6). Moreover they represent a possible diffuse source of contamination because they occur in the runoff from agricultural field irrigated with treated effluent [83]). Although the quantities of these contaminants found in groundwater are low, they represent a constant exposure source due to the fact that they are continuously present in the wastewater [16]. Their environmental fate and persistence, and the fact that they have been found in a multitude of animal tissues including human, make them cause of human health issues [16]. Galaxolide, due to the negligible photochemical degradation [10], could be introduced in the investigative monitoring due to the extensive and massive use as an indicator of urban setting contamination.

Among the OWCs coming from agriculture there are veterinary drugs. The use of veterinary drugs in intensively livestock represents the main route of their entry in the environment [6]. The extensive use of drugs-laden manure from the livestock industry on cropland also provides a route of entry for veterinary drugs into the groundwater system. Antibiotics are particularly serious environmental threat; their presence can cause the development of antibiotic resistance on humans due to the ingestion via animal or plant-based food products and the drinking of water with antibiotic residues. To date, little information is available on the occurrence and fate of veterinary antibiotics in groundwater considering that they are constantly released into the aquatic ecosystem. Sulfamethoxazole is, in general, the most widely reported antibiotics [60] (Table 4). In microcosm studies it is resulted to be a very persistent compound [58]. The presence of sulfamethoxazole could be attributed to the extensive use as veterinary antibiotics [112] and for human purposes [106]. The presence of the sulfamethoxazole could be related to the mixed agricultural-urban pressure (Tables 4–6).

The choice of these compounds between the different OWCs can be strategic because they are used in large doses and are found in groundwater. Carbamazepine is characterized by non-removal from the WWTPs and it could be found in the effluent at concentrations higher than at the input; the galaxolide instead is mostly removed from WWTPs. Moreover both molecules have been found in groundwater, underlining the different and complementary contamination pathways of groundwater bodies. The antibiotics sulfamethoxazole can be added to the investigative monitoring to understand the diffuse source contamination from mixed agricultural-urban pressure. The personal care and pharmaceuticals products considered in this study were at the highest levels of risk according to the PBT (Persistence, Bioaccumulation, and Toxicity) and OPBT total rankings (Occurrence, Persistence, Bioaccumulation, Toxicity; [79]).

Other OWCs can be used as markers for sewage intrusion like caffeine [98], [18], [38], [54], [53], [55], DEET [5], [62]; and the fecal sterols. [74], [87], [41].

The creation and further refinement of a conceptual model for OWCs in groundwater will be an iterative process. For that reason and because contaminants could have different origins, an integrated approach that combines the information coming from the investigative monitoring programme with the information coming from surface water, indicators and environmental tracers is required.

**Fig. 2:**
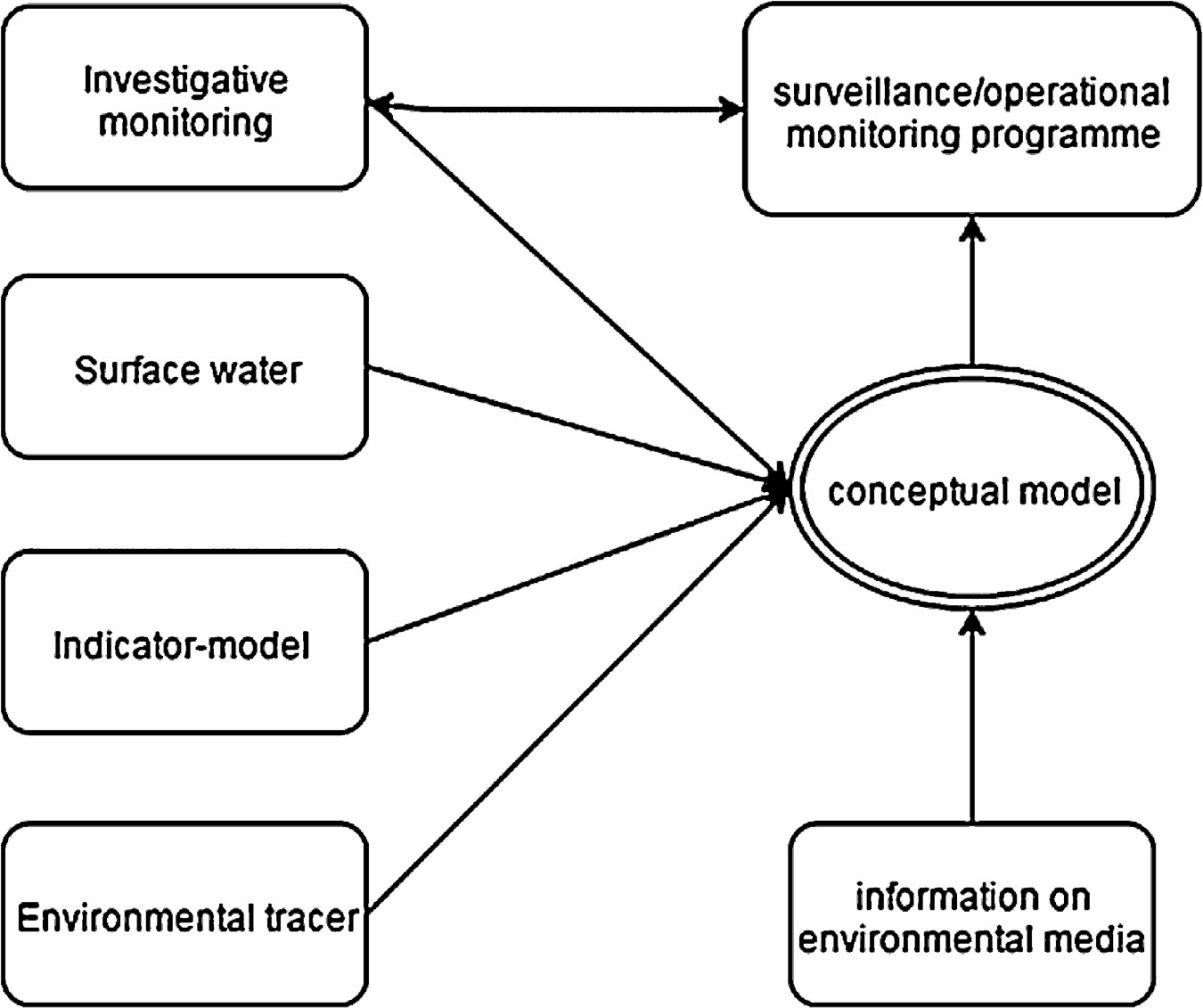
The proposed conceptual model derived by European Commission [35].

Information on monitoring point construction details, hydrological settings, aquifer type, understanding of recharge sources and patterns, local groundwater flow patterns and regimes, abstraction impacts, travel times and groundwater age distribution is very useful input to the development of the conceptual model [35] (Fig. 2).

## Conclusions

This paper demonstrates that the inclusion of emerging pollutants in monitoring programmes is feasible and expected by decision-makers and scientists. At present, these substances are not adequately considered in legislation, but there is a growing demands for the development of conceptual models representing emerging pollutant transfer from their sources to groundwater and its recipients, with the emphasis on ‘hot spot’ areas. In fact, to reach efficient water resource management the combination of regulation and management measures is required. Specific measures for the selected compounds could be useful to develop the conceptual model, according to the obtained results and to the existing knowledge on the field. Extensive and complete monitoring of groundwater is difficult to perform due to the high cost but the selection of specific environmental tracer could be useful to obtain information and to optimize benefit-cost analysis.

The effluents of the WWTPs are important point source of pollution of the groundwater [52]. OWCs are constantly released into the environment by human activities and are commonly detected in groundwater, but they are not sufficiently represented in conceptual models of groundwater systems: in fact modelling frameworks are well developed only for pesticides [42]

In the present paper the attention was focused on the case of OWCs using simplified pathways: from households to WWTPs to rivers and to groundwater; from bio solids, greywater, and livestock manure to soils and groundwater.

Because of consumer demands for safety and the lack of conceptual models for emerging pollutants in groundwater, policy-makers and scientists will have to cooperate for the creation of an initial groundwater emerging pollutant priority list. Some literature already exists and the selection of ranking models from existing and potential models could be based on the overall ranking proposed able to couple complexity, information and analytical technique availability and protection goals. The key factors for the compilation of the list of these compounds of interest are chemical properties, environmental exposure, toxicity, and occurrence information given by existing literature. The proposed molecules that could be added in this list are carbamazepine, galaxolide and sulfamethoxazole. The first two are molecules found in groundwater and originating by effluent of WWTPs, for which the study of the different pathways that can lead these molecules to groundwater is currently lacking. The understanding of their spatial and temporal occurrence should become a priority in order to develop the conceptual model. The third molecule is sulfamethoxazole, an antibiotic ubiquitously and persistent that can be related to both agriculture and urban pressure.

Integrated monitoring will contribute cost-effectively to the development of the conceptual model, enabling further surveillance and operational programmes.

## Figures and Tables

**Table 1:** Main organic wastewater contaminants.

Compound group	Compound class
Pharmaceuticals	Veterinary & human antibiotics; analgesics, anti-inflammatory & anti-histamine drugs; psychiatric drugs; lipid regulators; β-blockers & antihypertensives; X-ray contrasts; steroids & hormones; blood-viscosity affecting agents; antidiabetics; antidepressants; abuse drugs; stimulants
Personal-care products	Fragrances; sun-screen agents; insect repellents; antiseptics, biocides; moth repellents; surfactants
Pesticides	Insecticides, fungicides, herbicides, nematocides, biocides
Food additives	Antioxidants, sweeteners
Manufacturing additives	Corrosion inhibitors; flame retardants; gas propellants, plasticisers, plastic additives; stain repellents; surfactants, antioxidants, solvents, paraffin
Biocides	Biocides

**Table 2:** Approaches for making a priority list.

			Exposure						Toxicity		Other		
		Chemical properties	Consumption/Use	Fate in humans	WWTP efficiency	Predicted conc in surf.wtr	Measured conc in surf. wtr	Predicted conc in soil	Human	Environment	LCA	Literature occurrence	Multi-criteria
[27]	OWC	●					●						
[79]	Pharmaceuticals	●	●	●		●			●	●			
[51]	Emerging Pollutants	●	●	●		●			●	●			
[65]	Pesticides	●	●										
[84]	Pharmaceuticals	●	●	●	●	●							
[13]	Endocrine disruptors								●				
[17]	Pharmaceuticals	●				●			●	●			
[97]	OWC	●					●		●				
[34]	Storm water priority pollutants	●					●	●		●			
[19]	OWC	●	●					●	●	●			
[99]	Pesticides	●	●				●						
[95]	Pharmaceuticals					●				●			
[77]	Industrial chemicals	●							●	●			
[21]	Pharmaceuticals	●	●				●		●	●			
[33]	Pharmaceuticals	●	●	●	●	●			●	●			
[24]	Pharmaceuticals											●	
[75]	Pharmaceuticals and Personal Care products	●		●	●	●			●	●	●		
[56]	Pharmaceuticals and Personal Care products	●	●	●	●	●			●	●			●
[2]	Domestic substances	●	●			●			●	●			●
[46]	Industrial chemicals	●	●			•			●	●			●

**Table 3:** Selection of environmental tracers.

% of positive sampling GW (ranking)	Reference	Maximum concentration GW (ranking)	Reference	Toxicity classes	Removal from WWTP	Proposed overall ranking
Carbamazepine	[43]	Sulfamethoxazole	[62]	1-H benzotriazole (1)	1-H benzotriazole (L)	1-H benzotriazole
Galaxolide	[64]	DEET	[5]	Acetophenone (1)	Azhitromycin (L)	DEET
Galaxolide	[103]	NPE1C	[62]	Caffeine (1)	Carbamazepine (L)	NPE1C
Caffeine	[105]	Acetophenone	[5]	Cotinine (1)	Methylbenzotriazole (L)	Caffeine
BHT-CHO	[38]	Bisphenol A	[5]	Methylbenzotriazole (1)	PFOA (L)	Methylbenzotriazole
1-H benzotriazole	[53]	BHT	[38]	PFOA (1)	PFOS (L)	Carbamazepine
DEET	[62]	Sulfamethoxazole	[5]	PFOS (1)	Sulfamethoxazole (L)	Galaxolide
BHT	[38]	1-H benzotriazole	[62]	Tonalide (1)	Tris(2-chloroethyl)phosphate (L)	Sulfamethoxazole
Caffeine	[105]	Tris(2-chloroethyl)phosphate	[5]	Tris(2-chloroethyl)phosphate (1)	Galaxolide (M)	Bisphenol A
PFOA	[62]	Hydroclorotiazide	[105]	Azhitromycin (2)	Sulfamethoxazole (M)	1-H benzotriazole
1-H benzotriazole	[62]	BHT-CHO	[38]	BHT (2)	Acetophenone (H)	Tris(2-chloroethyl)phosphate
Methylbenzotriazole	[62]	Methylbenzotriazole	[62]	BHT-CHO (2)	Bisphenol (H)	Acetophenone
2-ethylhexyl 4-methoxycinnamate	[105]	Caffeine	[105]	Bisphenol A (2)	Caffeine (H)	PFOS
PFOS	[62]	DEET	[62]	Carbamazepine (2)	Cotinine (H)	Sulfamethaxine
Carbamazepine	[12]	Sulfamethaxine	[105]	DEET (2)	DEET (H)	PFOA
Carbamazepine	[62]	Carbamazepine	[62]	NPE1C (2)	NPE1C (H)	Hydroclorotiazide
NPE1C	[62]	4-AAA	[105]	2-ethylhexyl 4-methoxycinnamate (3)	Tonalide (H)	Azhitromycin
DEET	[5]	Galaxolide	[105]	Galaxolide (3)		Cotinine
PFHxS	[62]	Caffeine	[43]	Hydroclorotiazide (3)		Tonalide
Sulfamethaxine	[105]	Azhitromycin	[45]	Sulfamethaxine (3)		2-ethylhexyl 4-methoxycinnamate
Carbamazepine	[114]	Carbamazepine	[64]	Sulfamethoxazole (3)		4-AAA^a^
4-AAA	[105]	PFOS	[62]	Sulfapyridine (3)		BHT^a^
PFHpA	[62]	2-ethylhexyl 4-methoxycinnamate	[105]	4-AAA (3)		BHT-CHO^a^
Bisphenol A	[5]	Carbamazepine	[105]	PFDA (3)		PFDA^a^
Tris(2-chloroethyl)phosphate	[5]	Sulfapyridine	[105]	PFHpA (3)		PFHpA^a^
Carbamazepine	[105]	Cotinine	[11]	PFHxS (3)		PFHxS^a^
Sulfamethoxazole	[62]	1-H benzotriazole	[53]			Sulfapyridine^a^
Sulfapyridine	[105]	Carbamazepine	[12]			
PFDA	[62]	Tonalide	[91]			
Acetophenone	[5]	Galaxolide	[103]			
Sulfamethoxazole	[5]	PFOA	[62]			
Hydroclorotiazide	[105]	Carbamazepine	[114]			
Azhitromycin	[45]	PFHpA	[62]			
Cotinine	[11]	PFHxS	[62]			
Tonalide	[91]	PFDA	[62]			

Underlined compounds are the proposed environmental tracers.

a Indicates compounds with missing data.

**Table 4:** Carbamazepine, Galaxolide and Sulfamethoxazole occurrence information for groundwater (GW).

Compound	Country	GW mean or range ng/L^a^	GW max ng/L^a^	GW Frequency of detection %^a^	Reference
Carbamazepine	USA	40	420	1.46	[40]
	Europe	12	390	42	[62]
	UK	–	3600	–	[102]
	USA	–	–	20	[5], [39]
	Germany	2–900	–	–	[102]
	Germany	–	35	33	[114]
	France	–	10.4	–	[102]
	France	<10–100	–	14.3	[63]
	Spain	136	–	92–100	[64]
	Spain	–	62.4	48	[12]
	Serbia	3,4	–	17	[86]
Galaxolide	Germany	260	–	–	[59]
	Germany	4	17	–	[80]
	Spain	–	42.9	100	[103]
Sulfamethoxazole	Europe	–	38	24	[62]
	Germany	–	410	10	[94]
	France	–	18	18	
	USA	–	110	23.4	[5]
	China	–	250	93	[66]

a Stands for not available data.

**Table 5:** Carbamazepine, Galaxolide and Sulfamethoxazole occurrence information for surface water (SW).

Compound	Country	SW mean or range ng/L^a^	SW max ng/L^a^	Reference
Carbamazepine	World	174.2	11,561	[50]
	UK	0.5–251	684	[85]
	Italy	–	345	[69]
Galaxolide	Italy	<0.05–1141	1141	[109]
	Uk	28	28	Sunner et al. (2010)
	Romania	172–313	313	[72]
	Germany	40–1810	1810	[80]
	USA	45–794	794	[16]
Sulfamethoxazole	Hong-Kong	1.2	3.1	[26]
	Australia	8	2000	[111]
	France	nd–544	–	[104]
	Spain	13–149	–	[8]

a Stands for not available data.

**Table 6:** Carbamazepine, Galaxolide and Sulfamethoxazole occurrence information for biosolid.

Compound	Country	Mean biosolid conc. mg/kg dry wet	Reference
Carbamazepine	Spain	0.08	[89]
	Canada	0.26	[70]
	Canada	0.18	[44]
	Canada	0.09	[93]
	Spain	0.03	[73]
	Canada	0.01	[101]
Galaxolide	USA	177	[108]
	Canada	24.8	[115]
Sulfamethoxazole	Spain	84.4	[81]
	China	3.9	[61]
